# Assessing the precision of a time-sampling-based study among GPs: balancing sample size and measurement frequency

**DOI:** 10.1186/s12960-017-0254-8

**Published:** 2017-12-04

**Authors:** Daniël van Hassel, Lud van der Velden, Dinny de Bakker, Lucas van der Hoek, Ronald Batenburg

**Affiliations:** 1 NIVEL, Netherlands institute for health services research, P.O. Box 1568, 3500 BN Utrecht, The Netherlands; 2 CAOP, P.O. Box 556, 2501 CN Den Haag, The Netherlands; 3 Tranzo, Scientific Centre for Transformation in Care and Welfare, Tilburg University, P.O. Box 90153, 5000 LE Tilburg, The Netherlands; 4 Department of Sociology, Radboud University Nijmegen, P.O. Box 9104, 6500 HE Nijmegen, The Netherlands

**Keywords:** Time sampling, SMS text messaging, Confidence interval, Sample size, Working hours, Health workforce planning, General practitioners

## Abstract

**Background:**

Our research is based on a technique for time sampling, an innovative method for measuring the working hours of Dutch general practitioners (GPs), which was deployed in an earlier study. In this study, 1051 GPs were questioned about their activities in real time by sending them one SMS text message every 3 h during 1 week. The required sample size for this study is important for health workforce planners to know if they want to apply this method to target groups who are hard to reach or if fewer resources are available. In this time-sampling method, however, standard power analyses is not sufficient for calculating the required sample size as this accounts only for sample fluctuation and not for the fluctuation of measurements taken from every participant. We investigated the impact of the number of participants and frequency of measurements per participant upon the confidence intervals (CIs) for the hours worked per week.

**Methods:**

Statistical analyses of the time-use data we obtained from GPs were performed. Ninety-five percent CIs were calculated, using equations and simulation techniques, for various different numbers of GPs included in the dataset and for various frequencies of measurements per participant.

**Results:**

Our results showed that the one-tailed CI, including sample and measurement fluctuation, decreased from 21 until 3 h between one and 50 GPs. As a result of the formulas to calculate CIs, the increase of the precision continued and was lower with the same additional number of GPs. Likewise, the analyses showed how the number of participants required decreased if more measurements per participant were taken. For example, one measurement per 3-h time slot during the week requires 300 GPs to achieve a CI of 1 h, while one measurement per hour requires 100 GPs to obtain the same result.

**Conclusions:**

The sample size needed for time-use research based on a time-sampling technique depends on the design and aim of the study. In this paper, we showed how the precision of the measurement of hours worked each week by GPs strongly varied according to the number of GPs included and the frequency of measurements per GP during the week measured. The best balance between both dimensions will depend upon different circumstances, such as the target group and the budget available.

## Background

Insight into the working hours of doctors is of great importance for health workforce planning. How many hours doctors actually spend working, with their patients or not, is critical for assessing how many of them are available now and will be needed in the future. This applies, in particular, to general practitioners (GPs) who function as the gatekeepers to care in many health care systems. Thus, the working hours of GPs need to be monitored in order to ensure that primary care remains accessible [1]. Given their workload, it is essential to apply a method to measure their working hours which offer the least possible hindrance to GPs’ work but one which is also reliable.

Most studies investigating the working time of health care professionals are based on surveys, diaries or observations. In surveys, participants are asked to report their average time spent working per week over a certain period [2]. This is a quick and easy way to conduct research among large populations, but a significant disadvantage is the inaccuracy due to the impact of a bias towards overestimation or social desirability [3].

Diaries are considered as a more reliable research technique as they provide a more systematic way of measuring working time and a limited period of recall [4]. Diaries have been applied to measure the working time of doctors in several countries. For instance, Kmietowicz [5] obtained data from 329 GPs in the United Kingdom, using a daily schedule of working time, with activities structured in advance, during 1 week. And yet, for diaries, recall bias and errors are certainly not ruled out and they often impose a great burden upon the participants who have to write down their use of time for different activities on a daily basis.

Finally, observations are considered as the most reliable method. Two widely accepted techniques are “time and motion” and “work sampling”. With a time and motion study, an observer measures precisely how much time a participant spends on a specific activity. This is known as an accurate but also time-consuming technique, because the researcher is constantly observing the participant [6]. For this reason, this technique is often applied among smaller groups of respondents which raise doubts about to what degree their results can be applied generally [7]. Another limitation is that the presence of the observer can alter work behaviour, known as the Harwthorne effect [8, 9].

Work sampling is an alternative observation technique which involves an observer logging the activity of the participant at random moments [10]. This technique is far less time consuming than the time and motion technique and can result in comparable outcomes [11]. It appears difficult, however, to apply this among GPs who may possibly be working at any moment of the week, including evenings, nights and weekends. A more adequate format of work sampling, which may be applied to GPs or other target groups who work out of office hours, is time sampling [12]. Here, the participants themselves operate as the observer. They log their activities in real time at random selected moments, often with a self-reporting device, for instance beepers [13], PDAs [14, 15] or even smartphone apps [16].

Based on the principles of time sampling, we conducted an extensive survey of time use among Dutch GPs who were questioned about their activities at random moments during 1 week. A critical asset compared to other methods is reducing the potential of recall bias, because GPs were reporting about their activities in real time. Several instruments for performing this time-sampling technique were possible and considered, but we decided to use SMS text messages to perform the measurements of time use. Although SMS is assessed as relatively outdated, its underlying technology is highly standardized, it is accessible and its use is straightforward for all types of users. Currently, in Western countries, almost all citizens including doctors have a mobile phone at their disposal and are able to send SMS text messages [17]. To the best of our knowledge, SMS tools have never been applied to GPs to measure their working time.

The method provided a real-time measurement of the working hours of GPs and offered positive results regarding its feasibility for the respondents. In an earlier report, these results and the design of the study based upon time sampling and conducted with an SMS tool were discussed in detail [18]. The present study builds on this report and is focused on the impact of sample size and measurement frequency on the precision of the estimated working hours.

### Balancing precision and feasibility in the design of the method based upon time sampling

An important consideration in the use of a time-sampling method is the need to achieve a balance between the number of measurements for each participant and the number of participants. A higher number of measurements per participant means that fewer participants are required in order to obtain precise results. On the other hand, this decision will increase the workload for each participant and the probability too that they will drop out during the measurement period. We conducted a pilot study prior to our time-use study, which confirmed that it was feasible for GPs to send one SMS during every period of 3 h over all 7 days of 1 week [19]. In addition, GPs were able to indicate beforehand and on a daily basis if they were not working certain parts of a day, in particular, during evenings, nights and on weekends, thus avoiding the chance of these GPs receiving obsolete SMS text messages. With regard to how many GPs we should include in the study, we aimed to estimate the working time of six types of GPs as well, stratifying the required sample with regard to gender, self-employed, salaried and locum GPs. After investing much time and effort in recruitment, we succeeded in recruiting more than 1000 GPs for our time-use study. These participated fully during one of the weeks over a period of approximately 1 year.

The actual required sample size to attain a level of precision of working hours is an important issue for time-use researchers and health workforce planners who want to apply this method based on time sampling. This is particularly true if it concerns a study among GPs or other health care professionals who are harder to reach. It is also the case if fewer resources are available compared to our time-use study. In studies based on work or time sampling, however, there is hardly any information given about the sample size and the effect on the precision of the results. Even so, the technique is widely accepted and applied [20]. In a few cases, the confidence interval (CI) for the number of observations or measurements is reported [21, 22] and sometimes some general comments are made about the number of measurements needed [23, 6]. A more detailed analysis is important, because existing tools and power analyses to determine the required sample size are not sufficient if time-sampling studies are concerned. These tools only account for the uncertainty of the sample taken from a population while they do not account for the uncertainty resulting from the sample of measurements that is taken from a participant during a certain period. For this reason, prior to our study, we were not completely able to determine how many respondents and measurements we needed to attain a level of precision. The obtained data provides us a new and unique possibility to analyse this more thoroughly.

In the present paper, we address the question of to what extent the CI, the main precision indicator for the estimates of working hours, is related to the number of participants and the frequency of measurements per participant. To answer our research question, we used equations and conducted simulations on the time-use data among Dutch GPs as described above.

## Methods

### Data and materials

All GPs who participated during 1 week were questioned about their activities by sending SMS text messages. The message contained the single question: “What are you doing at this moment?” Included too was the time the message was sent in the format 00:00:00. Below this line, four possible categories of responses were listed on the SMS screen: (a) I am not working as a GP; (b) I am doing work directly related to my patients; (c) I am doing work indirectly related to my patients; and (d) I am not doing work related to my patients. Prior to the weeks of receiving SMS text messages, participants received an instruction including the definitions of the response categories.

SMS text messages were sent to the participants at random moments during 3-h time slots over a 24-h period. This implied that, per GP, eight messages per day and 56 messages per week, including the weekend days, were scheduled. In addition, so-called planning messages were sent at 7 am and 7 pm, by which GPs could indicate if they definitely would not work during a day part. If they used this option, their activities for these time slots were coded as “not working”.

Participants had to reply to every message which was pre-scheduled by entering their response letter and the order number of scheduled messages. The numbering started every day at 7 am. Numbering the SMS text messages was required to identify the specific message sent and to attach them to the activity answer of the participant to which it was related.

The data collection was conducted during 57 consecutive weeks over the period from December 2012 to January 2014. This was partly for logistic reasons, but it also enabled the researchers to account for seasonal variability in the data on working times which was collected. In total, 1051 GPs were included in the study of which 44 participated twice. In total, the participants represented about 9% of all the 11 075 GPs employed in the Netherlands in 2013. The composition of the response group regarding gender and position of employment corresponded to a large degree with the composition of the stratified sample that was drawn on the basis of these variables from the national registration of GPs conducted by NIVEL [24].

On average, 19 GPs participated for every week of the study of time use. More female (11) than male GPs (eight) were included, which corresponds with the distribution of gender within the sample. On average, 11 self-employed GPs participated. Furthermore, salaried GPs and GP locums were reasonably well represented in most of the weeks of the study.

Working hours were calculated by multiplying the replies to the activity questions with 3 as these were the time slots in which the messages were sent over the course of the week. For instance, the working week of a GP who replied that activity “b”, “c” or “d” was performed 13 times is calculated as 13 times 3 or 39 h. This seems to be a rough or inaccurate estimate of every GP’s working week. This is because the time measurements were taken only at samples of moments. However, as we included a large number of participants, this enables an accurate calculation to be made of the average working hours for the total group of GPs and its subgroups.

### Analyses

We used data from 1051 GPs for our extended analyses regarding the precision of the results. The first week of SMS text messages for the 44 GPs who participated twice was excluded from the analysis. We then analysed means, standard deviations and CIs for the working hours per week for all GPs and then the six subgroups. We used the total dataset of all GPs and measurements to explore the question of how the CI as a main indicator of precision varies, both for the number of participants and for the frequency of measurements for every participant.

Our data and analyses are based on sampling in two stages. Firstly, we analysed the sample of GPs out of the total population of GPs who were active in the Netherlands in 2013. Secondly, we analysed, for every GP, a random sample of moments of measurement in 3-h time slots during a week. As a result, the CI consists of two types of uncertainty. These are sample fluctuation and, secondly, moments of measurement fluctuation. The total CI was calculated through the following three steps.

#### Step 1: determining sample fluctuation

Sample fluctuation reflects the uncertainty of the sample of GPs drawn from the population. This is the conventional CI of an estimate that is commonly calculated for survey research.

We calculated the CIs for different numbers of GPs by adhering to the constant—that is the decision in our SMS study to ensure every participant received one message within 3-h time slots in the course of 1 week. This was done by using the standard deviation of the total measurements gathered from all GPs who participated. This standard deviation was weighted by the position of employment and gender of all GPs employed in the Netherlands in 2013. Then the following equation was applied:

$$ 95\%\hbox{-} \mathrm{CI}+/-=\left(\mathrm{weighted}\  \mathrm{standard}\  \mathrm{deviation}/\surd N\right)\times 1.96 $$

*N* represents the total number of measurements of different numbers of GPs and 1.96 is the *z*-value for the 95%-CI. The result is the one-sided divergent value, above or below, the average working hours within the total sample.

We then calculated the CIs for a varying frequent number of measurements per GP. We achieved this by multiplying the time measurements by 1.5 as if one measurement was taken every 2 h, by 3 as if one measurement was taken per hour and by 6 as if one measurement was taken every 30 min. Again, CIs were calculated for a varying number of GPs.

#### Step 2: determining measurement fluctuation

We simulated 1000 weeks of measurements taken from a fictional GP in order to gain insight into the uncertainty that result from the random points in time chosen for the measurements. We then calculated the one-tailed 95%-CI, the hours above or below the average value. This fictional GP works:

- 40 h per week
- only on weekdays from 7:30 am until 4:30 pm, with a 1-h break in the afternoon

We took this type of working week as analyses conducted previously had shown that most of the GPs were active at approximately these moments during the week [18]. Obviously, not all GPs will have this type of working week, but for the purpose of this analysis, it is expected that simulations based on this working schedule will provide most insight into the amount of measurement fluctuation that plays a role in the total CI. Additional simulations on other types of working weeks showed no substantial different results in most cases (Table 1).

**Table 1:** Additional simulations on different working schedules to calculate measurement fluctuation

To gain insight into the effect of a certain working schedule, we conducted additional simulations on other types of working weeks and calculated the total CIs according to step 3 which is described in this section. In these simulations, one measurement per half-, 1-, 2- and 3-h time slot during the week was assumed. The results were compared with the total CI based on the simulations on the 40-h working week we actually used in our analyses. This showed that the CIs would be a bit lower in most cases. The differences vary between − 0.21 and + 0.10 if 50 GPs are assumed and between − 0.12 and + 0.10 if 100 GPs are assumed (Table 4 in the Appendix).	

The simulations were performed taking 3-h time slots in the same manner as our SMS study. Subsequently, time slots of 2 h, 1 h and half an hour were simulated in order to investigate the effect on the precision of the results.

**Fig. 1:**
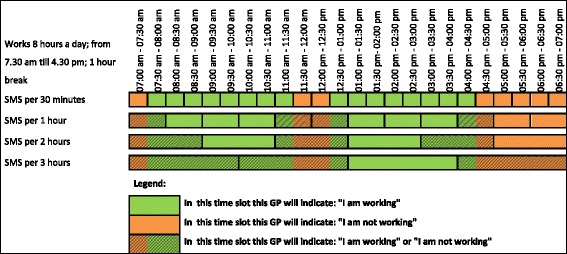
Moments on which a GP indicates I am working or not, with an SMS in different time slots. For a GP working on weekdays and from 7:30 am to 4:30 pm with a 1-h break in the afternoon. The beginning of the working day by a 30-min time slot was set at a couple of minutes from 7:30 am to simulate the CIs

Figure 1 illustrates how these simulations work. The green and orange parts of the figure reflect the periods in which the fictional GP, with this type of working week, will answer an SMS by replying “I am working”, answer (b), (c) or (d), and “I am not working”, answer (a). The shaded parts in the figure indicate the critical moments with regard to the accuracy of the measurements. This is because the response of the GP will depend upon the specific moment the SMS text message is sent within this time slot.

#### Step 3: Determining the total CI by combining sample and measurement fluctuation

We calculated the total CIs for a number of different measurement frequencies *and* for a varying number of GPs included in the dataset, based on the sample fluctuation (step 1) and measurement fluctuation (step 2). As both variances are independent of each other, we summed both types of uncertainty by applying the following equation:

$$ {\displaystyle \begin{array}{l}\mathrm{Total}\ 95\%\hbox{-} \mathrm{CI}+/-=\surd \left(\mathrm{weighted}\ \mathrm{SE}\ {\mathrm{sample}}^2+\mathrm{SE}\ {\mathrm{measurements}}^2\right)\times 1.96.\\ {}\end{array}} $$

This total one-sided CI is the key dependent variable that is analysed and presented in the next section of this paper. The two-sided CI will not be reported, but can simply be calculated by multiplying the one-sided results by factor 2.

The statistical analyses were performed in Stata 14.0.

## Results

### Precision of the GPs’ working hours based on our time-sampling study

Table 2 presents the average working hours, both for all GPs and for the six subgroups. These hours are based on the SMS measurements which were taken randomly once in every 3-h time slot according to the time-sampling technique. This design has resulted in almost 59 000 measurements. The average GP works 45.2 h per week. The hours above or below the mean with 95% confidence are relatively small. This shows that the actual values of the population are probably not much different from the results we measured. In most cases, the CI differs by 1 or 2 h from the average value. An exception is the male salaried GP, the smallest group of our sample, with a CI of 2.8 h.

**Table 2:** Means and one-sided CIs of GPs’ working hours, total and by position of employment and gender (based on the SMS measurements taken once every 3 h during 1 week for every GP)

	Number of measurements	Number of GPs	Mean hours	CI ± hours
Total^a^	58 856	1 051	45.18	0.67
Self-employed				
Male	16 296	291	51.91	1.32
Female	18 312	327	45.81	1.20
Salaried				
Male	3 136	56	41.57	2.81
Female	8 232	147	32.16	1.63
Locum				
Male	5 096	91	36.96	2.14
Female	7 784	139	33.13	1.68

The CI includes sample fluctuation and measurement fluctuation, as has been described in the “Methods” section

*CI* one-sided 95% confidence interval, hours above or below the mean

^a^Mean hours and CIs are weighted by position of employment and gender of the population of GPs

Considering the two types of uncertainty we explained in the previous section, it becomes clear that the largest part of the total CIs presented in Table 2 consist of sample fluctuation (not in the table). For example, the CI of the total sample of GPs is 0.60 h if only sample fluctuation is taken into account. This increases with 11% to 0.67 h if we include measurement fluctuation as well. Regarding the CIs of the six types of GPs, this increase varies between 10 and 14%.

### The CIs for an increasing number of GPs receiving SMS text messages during different time slots

**Fig. 2:**
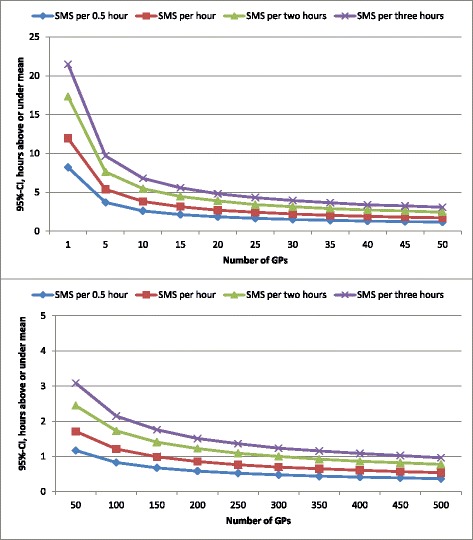
CI for one to 50 GPs and 50 to 500 GPs. The CI includes the weighted sample fluctuation and measurement fluctuation which is based on the equations and simulations on the data and is described in the “Methods” section. CI = one-sided 95% confidence interval, hours above or below the mean

What would be the CI if fewer GPs were included in the sample of our study and if more measurements per participant were taken? Figure 2 shows the one-sided 95%-CIs consisting of sample and measurement fluctuation, for an increasing number of GPs. We assume four different time slots for every measurement during 1 week. The CI for a 3-h time slot, according to our design, shows that the CI of the mean hours decreases from approximately 21 (one GP) to three (50 GPs) hours above or below the average value (upper part of Fig. 2). As could be expected, based on the formulas of calculating CIs presented in the “Analyses” section, the increase of the precision continues and is lower with the same additional number of GPs (bottom part of Fig. 2).

The same patterns are revealed if more measurements per GP were taken. Obviously, the CIs for these scenarios are lower compared to the CIs based on our design in which one measurement per 3-h time slot was conducted. For example, if one measurement per 3 h is taken for every participant, 300 GPs are needed to achieve a CI of 1 h. One measurement every hour requires just 100 GPs for the same result and one measurement every half an hour only 50 GPs.

## Discussion

We researched how a varied number of participants and measurements per participant have an impact upon the precision of the estimates of mean working hours per week, i.e. the one-tailed 95%-CIs. This was conducted on the basis of time-sampling data obtained by sending SMS text messages to 1051 Dutch GPs. The answer could support researchers and health workforce planners in determining the appropriate design and sample size required to obtain valuable time-sampling data about the working hours of GPs or other health care professionals.

Our results showed that the CIs of the mean working hours per week decreased from 21 h until approximately 3 h between the one and 50 GPs we included in the dataset and simulation. This was given a fixed number of time measurements per GP that was applied in our empirical study, i.e. once in every 3 h during the week. As a result of the formulas applied for calculating CIs, the impact of an increasing number of participants was smaller if a relatively high number of participants were already included. This means that proportionally a higher number of additional GPs were required for an increase of the precision.

Likewise, the number of participants required decreased if measurements were taken on a more frequent basis, for example once per hour instead of once per 3 h. Hence, the frequency of the measurements for every participant during the week is an important decision that determines the sample size required when this time-sampling technique is used. This is summarized by Table 3.

**Table 3:** Overview of estimates of participants needed and frequency of the measurements, with a certain CI

	Measurements during the week per:			
	Half an hour	Hour	2 h	3 h^a^
CI of ± 1 h	45	100	200	300
CI of ± 2 h	15	30	75	100
CI of ± 3 h	< 10	15	30	50

*CI* one-sided 95% confidence interval, hours above or below the mean

^a^One measurement per 3-h time slot was applied in our study

Considering these results, it is important to note that a standard is needed in order to claim what is the best level of precision required—the “optima”. The threshold for the minimal CI will depend on the purpose of a certain study. In our study, the working hours were needed for health workforce planning for which a certain precision is needed. However, like other key parameters in the model, some margin is possible as in most cases this will have no major impact on the advice regarding the number of GPs needed. Generally, we assess the CI cannot be larger than approximately 2 h, which implies that a maximum of 100 GPs are needed if at least one measurement per 3 h is taken from every participant during the week (second row of Table 3). On the other hand, a smaller CI would probably be required in studies in which the working hours are measured for other aims, like the remuneration of care. In these cases, a CI of 1 h could be necessary, which implies that a maximum of 300 GPs are needed assuming one measurement every 3 h (first row of Table 3).

In addition to the purpose of a study, there are several other circumstances that could play a role in deciding upon the appropriate sample size. This is, among others, the availability of the participants and the available time and resources of the researcher [25], the number of subgroups that have to be distinguished and the budget available to recruit and reimburse a target group for their participation. Additionally, researchers are dependent upon the willingness of a target group who will participate during the day. In any specific situation, different design choices could be considered. For example, if there are no different subgroups to account for, one could decide to conduct one measurement per 2 or 3 h during 1 week as this results in approximately 75 to 100 participants that are required for a reasonably precise estimate of working hours. This number of required participants increases considerably if there are subgroups or if a smaller CI is demanded. In response to this situation, the design could be adapted by increasing the number of measurements per participant. This could be done by extending the measurement period, for example by conducting measurements for every participant during 2 instead of 1 week. Another option, as we showed in our study, is to increase the number of measurements per participant during the week, i.e. one measurement per hour or more frequently. However, for researchers who want to apply the time-sampling method, it is important that this may then be less feasible for the participants and the risk of a low response rate, or participants dropping out altogether, would be greater. Instead of SMS text messaging, another device with which the participant could respond with minimal effort could then be considered.

### Limitations

Some limitations should be taken into account. Firstly, it was shown that the CI based on sample fluctuation increased with 11% if measurement fluctuation was taken into account. The measurement fluctuation was calculated by simulations of the data for participants during a specific 40-h working week. This should be assessed as an attempt to gain insight into this type of uncertainty as part of the CI, which is difficult to determine precisely. On the other hand, conducting these simulations on other working schedules resulted mostly in small differences with the total calculated CI. Further research is needed if a more detailed insight into the impact of measurement fluctuation is needed. This can be done by asking participants, prior to a week of measurement, to provide their working schedule on which simulations could be conducted separately.

Secondly, we relied on the accuracy of the GPs replies to the SMS text messages. Inaccurate reports may have biased the results. We believe this had only a limited impact as when we provided the respondents with an overview of their replies to the SMS text messages, we received only 10 to 20 emails with a few corrections. These were mainly regarding the type of activity which was not an issue in our analyses in this paper. Furthermore, 80% of all messages were replied to within 1 h which indicates a limited recall bias [18].

A third limitation of the analyses is that we focused only on the sample needed for measuring the total working hours as this is an important indicator for health workforce planning. However, in many studies, the work or time-sampling technique is applied in order to gain insight into a share of various different activities, expressed in percentages [6, 8, 10]. As Finkler and colleagues [6] have stated, this would probably imply that more measurements are needed, particularly when an activity is performed rarely. More research is needed for specific activities to gain insight into the samples needed for a certain degree of precision.

Finally, we used time-sampling data about GPs’ working hours obtained with an SMS tool that was assessed as a feasible and reliable technique. As we indicated in the introduction to this paper, different tools have been used in previous studies. These included beepers, PDAs or more recently smartphone apps [13–16]. These tools have benefits and drawbacks of their own, but it goes beyond the scope of this study to discuss these in detail. We recommend that these tools be taken into consideration if more measurements per participant are to be taken. For example, a small device with which the respondent can simply and quickly press a button to register an activity would be an alternative to SMS text messaging when a measurement is taken once every hour or more frequently.

## Conclusions

In an earlier study, we collected data on time use among Dutch GPs using a technique based on time sampling and an SMS tool. This appeared to be a valid method for measuring the number of hours GPs work each week and is an accessible and feasible research technique which can be applied to other target groups and countries. In the present paper, we have shown how the precision of the measurement of the hours worked by GPs each week varied according to the number of GPs included and the frequency of measurements per GP during the week measured. The best balance between both dimensions will depend upon different circumstances, among others the target group and the budget available.

## Appendix

**Table 4:** Total CIs based on simulations conducted on a 40-h working week and differences with the total CIs based on simulations conducted on six alternative working schedules, divided by the frequency of the measurements during 1 week, including 50 and 100 GPs

	Measurement per:			
	0.5 h	1 h	2 h	3 h
Total CI based on 40-h working schedule used (50 GPs)	1.17	1.71	2.44	3.09
Change of total CI in case of				
• Alternative 1	− 0.04	− 0.07	− 0.12	− 0.21
• Alternative 2	− 0.04	− 0.05	− 0.09	− 0.14
• Alternative 3	− 0.03	− 0.07	− 0.10	− 0.13
• Alternative 4	− 0.03	− 0.06	− 0.08	− 0.08
• Alternative 5	− 0.03	0.02	0.10	0.06
• Alternative 6	− 0.02	0.05	0.07	0.08
Total CI based on 40-h working schedule used (100 GPs)	0.83	1.21	1.73	2.15
Change of total CI in case of				
• Alternative 1	− 0.03	− 0.05	− 0.08	− 0.12
• Alternative 2	− 0.03	− 0.03	− 0.05	− 0.07
• Alternative 3	− 0.02	− 0.05	− 0.07	− 0.06
• Alternative 4	− 0.02	− 0.04	− 0.06	− 0.02
• Alternative 5	− 0.02	0.02	0.08	0.05
• Alternative 6	− 0.02	0.03	0.05	0.10

The total CI is based on the combination of sample and measurement fluctuation as has been explained in the “Methods” section. Simulations on a 40-h working schedule are conducted to assess the amount of measurement fluctuation. All working days start at 7:30 am. The beginning of the working day by a half an hour time slot was set at a couple of minutes from 7:30 am to simulate the CIs

Legend: Working schedule used in this paper = 5 working days of 8 h; 1-h break (40 h per week). Alternative 1 = 2 working days of 8 h; 1-h break (16 h per week). Alternative 2 = 3 working days of 8 h; 1-h break (24 h per week). Alternative 3 = 4 working days of 4 h; no break (16 h per week). Alternative 4 = 5 working days of 4 h; no break (20 h per week). Alternative 5 = 6 working days of 10 h; 1-h break (60 h per week). Alternative 6 = 7 working days of 8 h; 1-h break (56 h per week)

## Abbreviations

CI: Confidence interval
GP: General practitioner
